# Enhancing Double-Beam Laser Tweezers Raman Spectroscopy (LTRS) for the Photochemical Study of Individual Airborne Microdroplets

**DOI:** 10.3390/molecules24183325

**Published:** 2019-09-12

**Authors:** Jovanny A. Gómez Castaño, Luc Boussekey, Jean P. Verwaerde, Myriam Moreau, Yeny A. Tobón

**Affiliations:** 1 Univ. Lille, CNRS, UMR 8516-LASIR-Laboratoire de Spectrochimie Infrarouge et Raman, F-59000 Lille, France; luc.boussekey@free.fr (L.B.); Jean-Pierre.Verwaerde@univ-lille.fr (J.P.V.); myriam.moreau@univ-lille.fr (M.M.); 2 Laboratorio de Química Teórica y Computacional, Grupo Química-Física Molecular y Modelamiento Computacional (QUIMOL), Facultad de Ciencias, Universidad Pedagógica y Tecnológica de Colombia, Tunja, Boyacá 050030, Colombia

**Keywords:** laser tweezers Raman spectroscopy (LTRS), atmospheric aerosols, optical levitation, optical traps, photochemical reactions in Earth’s atmosphere

## Abstract

A new device and methodology for vertically coupling confocal Raman microscopy with optical tweezers for the in situ physico- and photochemical studies of individual microdroplets (Ø ≤ 10 µm) levitated in air is presented. The coupling expands the spectrum of studies performed with individual particles using laser tweezers Raman spectroscopy (LTRS) to photochemical processes and spatially resolved Raman microspectroscopy on airborne aerosols. This is the first study to demonstrate photochemical studies and Raman mapping on optically levitated droplets. By using this configuration, photochemical reactions in aerosols of atmospheric interest can be studied on a laboratory scale under realistic conditions of gas-phase composition and relative humidity. Likewise, the distribution of photoproducts within the drop can also be observed with this setup. The applicability of the coupling system was tested by studying the photochemical behavior of microdroplets (5 µm < Ø < 8 µm) containing an aqueous solution of sodium nitrate levitated in air and exposed to narrowed UV radiation (254 ± 25 nm). Photolysis of the levitated NaNO_3_ microdroplets presented photochemical kinetic differences in comparison with larger NaNO_3_ droplets (40 µm < Ø < 80 µm), previously photolyzed using acoustic traps, and heterogeneity in the distribution of the photoproducts within the drop.

## 1. Introduction

Optical tweezers (OT) are a state-of-the-art technique currently used in several research disciplines such as environmental, basic, pharmaceutical, and materials and engineering sciences for the study and manipulation of a wide variety of nano- to microscale samples captured by the forces exerted by lasers [1]. The particles studied using this technique range from biological systems such as microorganisms, cells, and organelles to physical materials, atmospheric, and medical aerosols, combustion and industrial emissions as well as chemical compounds and solutions. OT have been employed not only for the trapping of micrometric samples in solution, but also for the levitation of condensed phase particles in a gas or in a vacuum.

One of the disciplines in which OT has experienced great application and development in recent years is terrestrial climate studies. The study on the impact of individual aerosols in the atmosphere is of particular interest. By using OT, analyses such as: (a) phase behavior, hygroscopic growth, morphology, vapor pressure, and the kinetics of surface mass transference [2,3]; (b) whispering gallery mode (WGM) resonances [4] and other optical properties [5]; (c) heterogeneous oxidation reactions [6]; (d) isotopic exchanges [7]; (e) coalescence properties [8]; (f) in situ quantification of inorganic solutes [9]; (g) viscosity [10]; and (h) time-varying composition and oxidative aging [11], have been performed over the last decade on individual aerosols levitated in air.

In addition to OT, electrodynamic balances (EDB) and acoustic traps (ATs) are techniques that are also used for the levitation and study of individual aerosols [2]. All of these levitation techniques are usually combined with light-scattering spectroscopy to conduct nondestructive chemical composition and physical experiments on individual aerosols [2,12,13]. However, the techniques differ in the method used for the capture as well as the size of the isolated particle; therefore, these variables must be considered when selecting the type of system to be studied. For instance, with OT, particles between 1 and 10 μm in diameter are captured, while particles with diameters ranging from 5 to 50 μm or 20 to 100 μm are isolated using EDBs and ATs, respectively [2]. As most of the Earth’s atmospheric aerosols have a very small diameter (<5 μm), both EDBs and OT are the most suitable methods when considering size (i.e., the surface’s curvature) in the study of the physicochemical properties and dynamics in aerosols of environmental interest. A limiting factor of EDB is that only charged particles can be trapped.

Raman microscopy has been successfully coupled with OT for the spectroscopic characterization of both solution and airborne particles, giving rise to an optical hybrid technique that is better known as laser tweezers Raman spectroscopy (LTRS). The original development of LTRS coupling can be attributed to Thurn and Kiefer, who in the mid-1980s demonstrated the simultaneous capture and measure of Raman scattering of micrometric sized solid and liquid particles in air using single argon or krypton ion gas lasers weakly focused on the particle through conventional optical lenses [14,15]. Nowadays, LTRS instruments are equipped with high numerical-aperture (NA) microscope objectives, through which the laser beam of the tweezers can be strongly focused onto the particle. Such strong focusing allows for greater stabilization of the optically trapped particles by means of a significant photon moment transfer caused by the scattering and gradient forces, which act in the direction of propagation and the lateral plane of the incident laser, respectively [1]. A compendium of the different studies performed on optically trapped single particles in air has recently been reported by Gong et al. [16].

**Figure 1:**
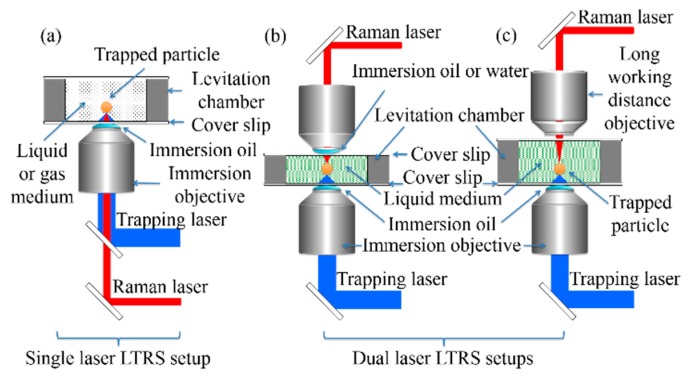
Schematic illustrations of the two main microscope objective coupling configurations currently used in laser tweezers Raman spectroscopy systems. (**a**) Single inverted immersion objective configuration, (**b**) two immersion objectives and (**c**) an inverted immersion objective combined with a long-working-distance objective.

By considering the number, type, and disposition of the microscope objectives used in LTRS couplings, two general types of beam configurations can be utilized. The most common configuration uses a single inverted microscope immersion objective (Figure 1a), through which one (for simultaneous trapping and Raman analysis) or two (one for trapping and one for Raman analysis) lasers can be focused. This kind of setup has been widely used for microbiology studies of single living cells [17,18,19,20,21,22,23] and bacteria [24,25,26,27,28,29] in aqueous solution as well as for physicochemical studies of polymer microspheres [30,31,32], carbon nanotubes [33], and small proteins [34] in liquid media. It has also been used to study individual microdroplets (aerosols) in the gas phase [4,6,8]. The second main configuration of LTRS uses two microscope objectives, one for capturing and one for Raman scattering. In this case, two variants are utilized: (i) a configuration with two immersion objectives (Figure 1b), in either the vertical [35] or horizontal [36] orientation; or (ii) a vertical arrangement with an inverted immersion objective and an upward long-working-distance (LWD) objective for Raman measurements [37] (Figure 1c). Although the use of two-objective LTRS instruments has been devoted to biological particles captured in liquid media such as single cells and protein aggregates [35,36,37], the availability in this setup of an extra objective, in addition to that used for capturing, will open the possibility of using confocal Raman microscopy to perform spectral mappings on airborne aerosols. This enables the possibility of carrying out in situ studies of the chemical distribution of its components. Some horizontal variations have been reported using two LWD objectives, through which two counter propagating hollow beams (CPHB)—produced by splitting a single laser trap beam—are focalized on particles levitated in air [38]. However, this type of configuration does not allow the change of Raman excitation lines and makes the implementation of confocal Raman microscopy impractical. Other configurations using fiber beam traps instead of microscope objectives have also been reported for the study of living cells in solution [39,40].

As far as we know, the double-objective LTRS configuration has not yet been used for in situ photochemical experiments or Raman imaging on airborne microdroplets [16]. Kalume et al. recently performed position-resolved Raman spectroscopy on an optically levitated diethyl phthalate single droplet (Ø ≤ 20 µm) [41]; however, this is still far from being a Raman imaging experiment. In order to bridge this instrumental gap, a new vertical coupling system that extends the current applicability of dual-laser LTRS systems to the levitation, Raman imaging, and photolysis experiments of individual aqueous microdroplets in air was designed and built. This system has allowed us to expand the photochemical studies that have recently been performed on particles between 20 and 80 μm in diameter, using acoustic levitation coupled with Raman spectroscopy [12,13] for smaller particles (Ø ≤ 10 µm). By combining these two techniques, the impact of size on photochemical reactions (heterogeneous and homogeneous) and the properties of the airborne aerosols will be better understood. This setup allows a variety of physico- and photochemical studies to be performed in situ, but also enables spatial Raman mapping on the levitated droplet. This provides detailed chemical information from the Raman spectra at each pixel by using the capabilities provided by confocal Raman microscopy. In addition, the system offers the advantage of changing the Raman excitation wavelengths without interrupting the levitation process, depending on the requirements of the experiment. In order to test the LTRS device and evaluate the impact that size has on the photochemistry of an aerosol of environmental interest, aqueous microdroplets containing sodium nitrate salt were selected. The photochemistry of these particles was recently studied by us in droplets of larger sizes (20 to 80 µm in diameter) using an AT device coupled to Raman microscopy [12,13].

## 2. Instrumental Setup

**Figure 2:**
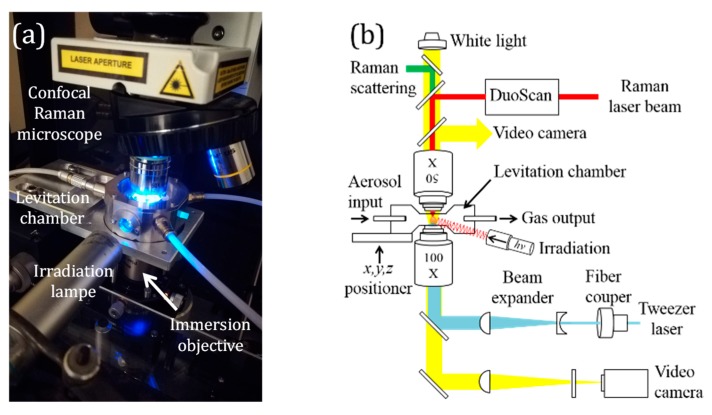
Experimental setup. (**a**) Photograph and (**b**) schematic diagram of the dual-objective LTRS system used in this study for the physico- and photochemical study of levitated aerosols in air.

A schematic representation of the LTRS coupling system is presented in Figure 2. The main component of the setup is a custom made portable photochemical levitation chamber that joins the inverted immersion objective of an optical tweezer system and the upward objective of a confocal Raman microscope. This setup merged the Nikon 100× immersion objective from an optical tweezer (Thorlabs™) with the Nikon LWD 50× objective of a LabRAM HR confocal Raman microscope (Horiba Scientific, France).

### 2.1. Optical Tweezers

The optical trap was a Modular Optical Tweezers OTKB(/M) (Thorlabs™, USA) equipped with a Nikon 100× oil immersion objective (NA = 1.30), a 975 nm trap laser (variable trapping power from 1 to 92 mW), and a DCU224 video camera (Thorlabs™, USA) (1280 × 1024 pixels) for imaging, and a 3-axis sample positioning stage.

### 2.2. Confocal Raman Microspectrometer

A LabRAM HR confocal Raman microscope (Horiba Scientific) equipped with 300, 600, and 1800 grooves/mm gratings, a back-illuminated liquid-nitrogen-cooled charge-coupled device (CCD) detector (1024 × 512 pixels), an Olympus 50× long-working-distance microscope objective (NA = 0.5, WD = 10.6 mm), and three excitation laser beams at 473, 532, and 633 nm with 18, 30, and 17 mW of power, respectively, were applied to the surface of the sample. Depending on the sample’s properties, the Raman laser power can be reduced by 0.01%, 0.1%, 1%, 10%, 25%, or 50% by using density filters. The Raman spectrometer also includes a video-camera for viewing and a DuoScan™ imaging system. The latter consists of a set of scanning mirrors that sends the laser beam precisely through a pattern predefined by the analyst.

In this study, Raman mappings of the levitated droplet were collected using the 600 groove/mm grating and the 532 nm laser (power on the droplet = 7.5 mW) in a point-by-point XY scanning mode using the DuoScan™ imaging system. A 9 × 9 μm region of interest containing the droplet was scanned using steps of 1 × 1 μm. Correct signal to-noise ratios were obtained with an acquisition time of 30 s with two accumulations by spectrum. Single Raman spectra of the droplets were also collected with the three Raman lasers to test the possibility of changing the Raman excitation during the experiment and in selecting the most adequate Raman line.

### 2.3. Photochemical Levitation Chamber

**Figure 3:**
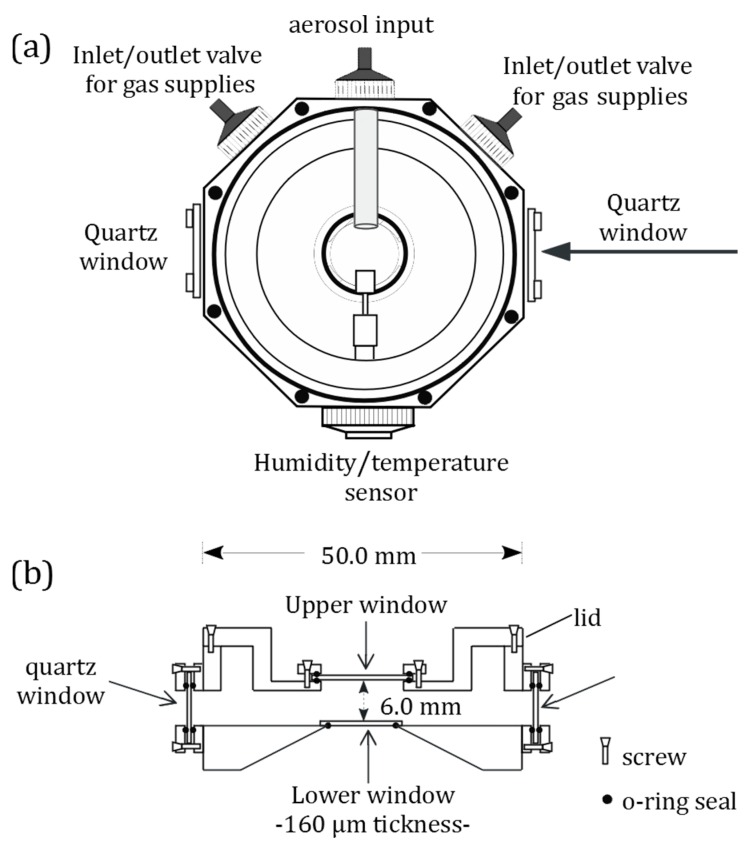
(**a**) Top (without lid) and (**b**) cross-section views of the levitation chamber used for coupling an inverted immersion objective of an optical tweezer and an LWD objective from a confocal µ-Raman microscope.

A schematic diagram of the designed levitation chamber is presented in Figure 3. The levitation chamber provides a sealed environment for the study of single particles suspended in air. Different atmospheric conditions can be reproduced and a wide variety of physical, chemical, and photochemical processes can be performed. This consisted of a custom made octagonal stainless-steel cell with a removable cover for easy access and cleaning of the interior. The sidewalls of the chamber were equipped with injection and exhaust ports, openings for different windows (e.g., quartz or glass) for photochemical measurements, and a temperature/humidity sensor. An aerosol injection port was precisely directed at the center of the device in order to trap particles. In the central part of the lower surface, the chamber has a circular hole that is covered from the inside with a coverslip (160 µm thickness), and treated with a surfactant solution (sodium dodecyl sulfate, SDS, 1 mM) prior to each levitation experiment to avoid droplet formation on the coverslip. The upper lid was equipped with an opening for a quartz or glass window for visualization and Raman image collection through the LWD microscope objective. The internal distance between the lower surface and upper lid was around 6 mm. All of the openings in the levitation chamber were air tight due to the use of O-ring seals. The coverslip was pressed against the bottom O-ring by two semicircular metallic shields as part of the removable cover. These shields limit the capture volume and concentrate the particles in the center of the cell near the trapping point.

### 2.4. Coupling of the Optical Tweezers with the Vertical Raman Microscope

Optical tweezers were placed directly under the Raman microscope. Optical alignment between the laser of the optical tweezer and the Raman spectrometer was achieved by means of a micrometric XY-adjustment adapted to the metal platform that supported the tweezer device. This was assisted by the visualization of the beam from the optical tweezer using the camera of the Raman microscope. The Z-adjustment of the working distance between the LWD objective and the levitation chamber was achieved using the Z-position adjustment knob of the Raman microscope. By using the XYZ micrometric positioning stage from the Thorlabs™ optical tweezers, fine tuning of the position of the cell could be performed so that the focus point was located a few tens of micrometers above the covered coverslip. Short-pass filters (FES0750 Thorlabs™ cut-off at 750 nm wavelength) were placed before the CCD detector to block the trap laser and the video camera of the Raman spectrometer as well as allow visualization of the levitated droplet without interference of the trap laser.

## 3. Results and Discussion

The LTRS coupling system was tested by conducting a Raman study of an aqueous microdroplet of NaNO_3_ levitated in air and then exposed to UV light (254 ± 25 nm). In Figure S1 (Supporting Material), one of the nitrate aerosols (approximately 8 μm in diameter) captured with this setup is shown. The image was simultaneously visualized from both the top (through the upward Raman microscope objective) and from the bottom inverted oil-immersion objective of the tweezer device. In addition, a video showing the process of particle capture in the optical trap using this system is also provided in the Supplementary Materials. The particle was highly stable when suspended, remaining in this state for more than a week. The levitation time of this particle could have been extended if the tweezer laser had not been turned off deliberately. Additionally, no change in the chemical composition of the particle was observed due to the trap laser.

Spectroscopic characterization of the optically levitated NaNO_3_ droplet was performed by focusing the excitation laser through the DuoScan^TM^ device of the Raman confocal microscope. The Raman spectra were collected using excitation wavelengths of 473, 532, and 632 nm. The Raman spectra showed the three expected Raman-active bands (ν_1_, ν_3_, and ν_4_) for a planar NO_3_^−^ ion (*D_3h_* symmetry) at ~1064/1411/731 cm^−1^, ~1049/1414/710 cm^−1^, and ~1053/1401/715 cm^−1^, using the excitation lines 473, 532, and 632 nm, respectively (see Figure S2 in the Supporting Material). These frequencies were similar to those obtained in previous studies using larger particles (40 µm < Ø < 80 µm) by means of the acoustic levitation technique [12,13], and the reported values for supported NaNO_3_ microdroplets [42]. Further spectra were measured using the excitation wavelength of 532 nm due to its better signal-to-noise ratio.

**Figure 4:**
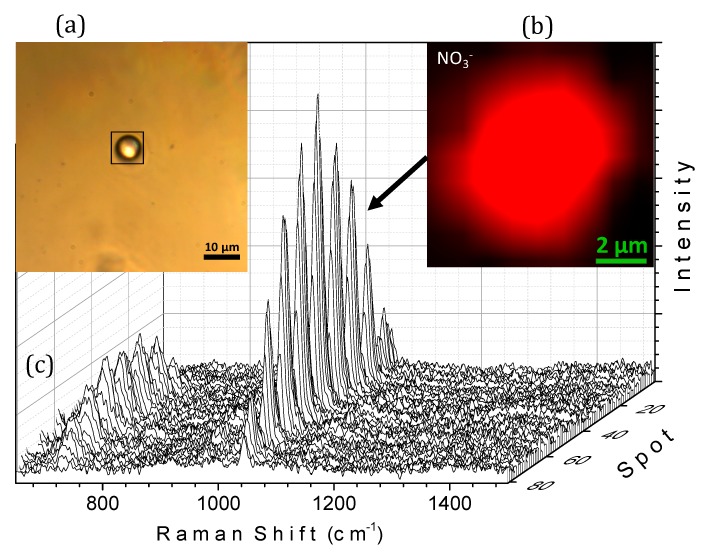
Optically levitated droplet containing aqueous NaNO_3_ before irradiation. (**a**) optical image showing the region of interest defined by the black frame; (**b**) Raman image of the region of interest reconstructed from the 1020–1070 cm^−1^ spectral range after base line correction and smoothing procedure; (**c**) Raman spectra of each spot, recorded using an excitation laser at 532 nm.

To evaluate the ability of the device to capture Raman images and spatially resolved spectroscopic information, micro-Raman mapping in the XY plane with the DuoScan^TM^ device was performed. A 9 × 9 μm region of interest containing an aqueous NaNO_3_ particle was scanned using 1 × 1 μm steps. Figure 4 demonstrates the 81 spectra obtained from this two-dimensional map and the Raman image reconstructed from the region of interest using the NO_3_^−^ symmetric stretching mode near 1049 cm^−1^. As expected, the NO_3_ ion was homogeneously distributed throughout the droplet.

**Figure 5:**
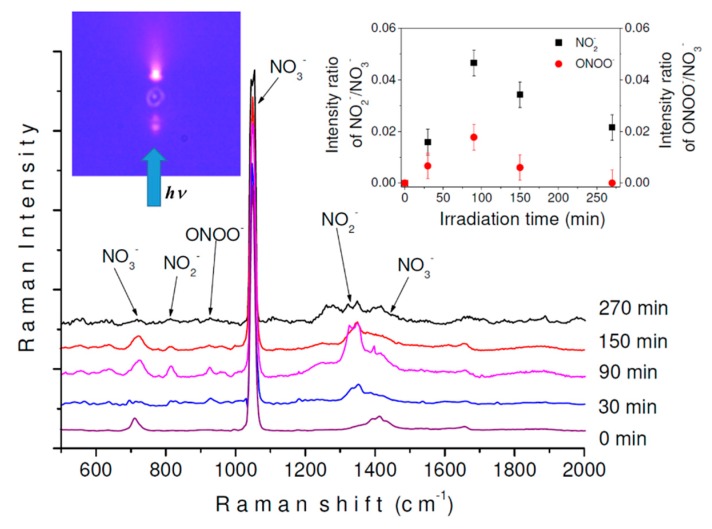
Raman spectra of a microdroplet of aqueous NaNO_3_ obtained after different times of UV light irradiation (254 ± 25 nm) using an excitation line of 532 nm. (**Left inset**) Photograph of top capture of the particle during the irradiation process. (**Right inset**) Intensity of NO_2_^−^/NO_3_^−^ and ONOO^−^/NO_3_^−^ ratios as a function of the irradiation time.

After optical trapping and Raman analysis of the single NaNO_3_ particle, the droplet was exposed to a collimated UV beam (254 ± 25 nm). Changes were monitored by recording the Raman spectrum as a function of the irradiation time. As the diameter of the UV spot from the lamp was larger than the size of the drop, the particle was completely covered by the radiation beam, thus ensuring full irradiation of the droplet. The photograph within Figure 5 provides a visualization of the particle during irradiation exposure, where the blue arrow indicates the direction of the lamp’s beam. The droplet is illuminated when the UV beam goes through it and some reflection and transmission of light can be observed in the picture. To the best of our knowledge, this is the first time that a photochemical study with Raman mapping has been performed on an optically levitated airborne microdroplet. The development of this instrument was primarily due to experimental difficulties associated with the design and construction of coupling LTRS cells.

Figure 5 also shows the Raman spectra of the droplet initially containing an aqueous solution of NaNO_3_ and after 30, 90, 150, and 270 min of exposure to UV light. Each Raman spectrum was collected with the DuoScan™ system using an averaging mode across a predefined circular area corresponding to the droplet’s diameter (8 µm for the droplet in Figure 5). After 30 min of irradiation, new bands were observed at 1337, 814, and 1262 cm^−1^, similar to the ν_1_, ν_2_, and ν_3_ vibrational modes of the NO_2_^−^ ion reported at 1330, 820, and 1243 cm^−1^ in bulk aqueous solution [43], respectively, and observed at 1330, 815, and 1278 cm^−1^ for the photochemically formed ion in a microdroplet using acoustic levitation [12]. A new feature at 914 cm^−1^ was also observed with irradiation, which was identified as the bending mode of peroxynitrite ion, ONOO^−^ [12,44].

As shown in the plot of the intensity ratio of NO_2_^−^/NO_3_^−^ and ONOO^−^/NO_3_^−^ as a function of irradiation time (inset in Figure 5), both photoproducts showed similar behavior with irradiation time. These were increased until a maximum concentration of NO_2_^−^ and ONOO^−^ ions in the droplet was observed after 90 min of irradiation. When photolysis reached 270 min, ONOO^−^ disappeared while the NO_2_^−^ band intensities decreased. Detection of the NO_2_^−^ ion as the final photoproduct in this experiment is in line with the overall photolysis reaction NO_3_^−^ → NO_2_^−^ + O_2_ (formed from O^•^ radicals) reported for nitrate in an aqueous medium using λ ≥ 195 nm [45,46]. In the present study, ONOO^−^ was consumed after 270 min of irradiation, and the NO_2_^−^ was decreased, but not completely eliminated. This is similar to other studies where ONOO^−^ was identified as an intermediate in the formation of nitrite by photoisomerization of NO_3_^−^ at λ < 280 nm [45,47]. However, some background fluorescence was observed to increase in the Raman spectra beyond 90 min, and observed intensities could have overlapped.

The behavior of the products generated by the photolysis in a droplet 1/10 the size, is quite different from previous observations for the photolysis of a larger drop of NaNO_3_ using acoustic levitation [12]. In a larger acoustically levitated droplet, no changes were observed after 2 h of irradiation, probably due to a self-inhibited surface process. Consequently, droplet size (i.e., curvature surface) plays an important role in the behavior of the products with irradiation time. Thus, these results complement those obtained in larger particles and bulk aqueous solutions.

**Figure 6:**
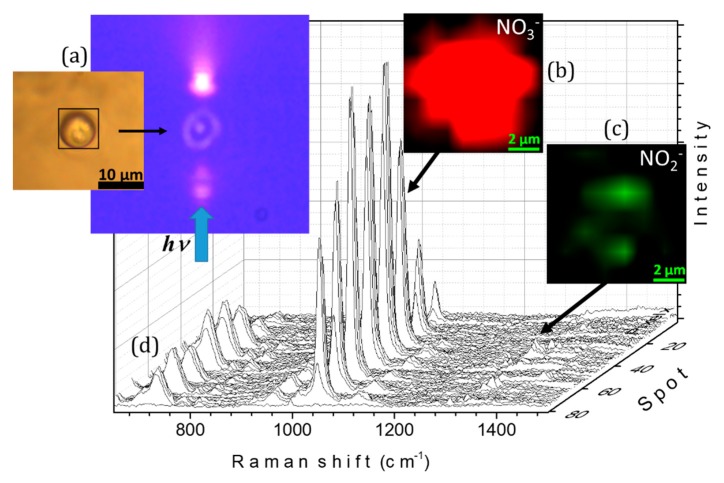
Optically levitated droplet initially containing aqueous NaNO_3_ after 270 min of 254 ± 25 nm irradiation (**a**) optical images showing the region of interest defined by the black frame and the particle during the irradiation process and (in blue) the direction of the irradiation beam; (**b**) and (**c**) Raman images of the region of interest reconstructed from the 1020–1070 and 1300–1350 cm^−1^ spectral ranges, respectively, after base line correction and smoothing; (**d**) Raman spectra of each spot recorded using an excitation laser of 532 nm.

After 270 min of irradiation, micro-Raman mapping was also performed to obtain spatially resolved spectroscopic information and evaluate the distribution of the NO_3_^−^ and NO_2_^−^ products in the particle (see Figure 6). The Raman images with the 9 × 9 μm region of interest containing the droplet were reconstructed using the symmetric stretching modes near 1049 and 1337 cm^−1^ of NO_3_^−^ and NO_2_^−^, respectively. The images revealed the heterogeneous distribution of nitrite ion photoproducts in the irradiated particle, mainly concentrated in two discrete areas of the droplet (Figure 6c). These areas correlate with the direction of the irradiation beam (Figure 6a). Our hypothesis is that the formation of NO_2_^−^ in localized areas is due to the distribution of light within the drop. The fact that the distribution of NO_2_^−^ within the droplet remains heterogeneous during the measurement of the Raman spectrum signifies a very low agitation of the liquid medium, hence slow NO_2_^−^ diffusion within the levitated particle at room temperature.

## 4. Materials and Methods

### 4.1. Sample Preparation and Levitation

Commercial sodium nitrate (Alfa Aesar, 99.999%) and ultrapure deionized water (Milli-Q, 18 MΩ) were used to prepare an aqueous solution of NaNO_3_ (3 M). Droplets were optically trapped by introducing a dispersion of aqueous NaNO_3_ aerosol generated by a medical ultrasonic nebulizer (Omrom MicroAIR U22, Japan) into the levitation chamber through the injection port. Particles between 5 and 8 μm in diameter were trapped by the gradient and scattering forces generated at the focal point of the laser tweezer. A bubbler was installed to maintain a relative humidity of around 80% during the entire experiment with the experiment being performed at room temperature.

### 4.2. Photolysis of Levitated Particles

Levitated droplets were exposed to a collimated beam (beam spot diameter = 6 mm) from a Hg–Xe arc lamp, Hamamatsu, LC8 Lightningcure, 200 W (Hamamatsu, Japan), equipped with a bandpass filter (Semrock^TM^, USA) centered at 254 ± 25 nm (power on the droplet = 4.07 × 10^−3^ µW/µm^2^). Composition of the droplets, before and after photolysis, was monitored by micro-Raman spectroscopy (Horiba Scientific, France).

## 5. Conclusions

An alternative vertical dual-laser coupling for laser tweezers Raman spectroscopy (LTRS) was presented. The coupling system is a prototype of a fully functional photochemical levitation chamber, which allows the correct coupling between a commercial optical tweezer, equipped with an inverted immersion objective, and a micro-Raman spectrophotometer equipped with a vertically descending LWD objective and the DuoScan^TM^ system. This new configuration offers the possibility of performing in situ photochemical experiments as well as easily and conveniently exchanging the different Raman excitation lines, without interrupting the levitation of the particle or moving the upper objective. This study is the first in reporting photochemical studies and Raman mapping on optically levitated droplets. In addition, this study demonstrated that spatially resolved spectroscopic information can be obtained from a single particle before and after physico- or photochemical processes. The effectiveness of the system was tested by studying the photochemical properties of microdroplets of atmospheric interest containing inorganic salts, in this case, sodium nitrate. The Raman images allowed for the characterization of the heterogeneity of the nitrite ion photoproduct of a single optically levitated particle, with a lateral resolution of 1 µm^2^. Particle agitation was limited in our experiment because homogenization of the photoproduct did not occur within the collection time. It was also demonstrated that using an optical trap instead of acoustics is useful to mimic size-dependent processes.

One of the main sectors in which this device and technique can be applied is in atmospheric physical chemistry research. By using this system, aerosols of atmospheric interest can be studied. With this technique, it is possible to study not only the physicochemical properties such as morphology, optical activity, hygroscopicity, vapor pressure, reactivity, and chemical composition, but also the photochemical behavior of atmospheric aerosols. Marine, urban, forest, industrial, and volcanic aerosol proxies are some examples of particles of atmospheric relevance that can be studied with this device. The distribution of different compounds on the surface of a micrometric particle (heterogeneity) can be studied without the influence of a contacting surface. Likewise, a wide variety of heterogeneous chemical or photochemical reactions can be performed using this device, for instance, photocatalytic oxidative reactions occurring on the surface of the droplet could be studied by introducing a reactive gas into the levitation chamber. Other relevant systems such as medical or fungicide aerosols can also be studied.

## Supplementary Materials

The following are available online, Figure S1: Raman spectra of a micro-droplet of aqueous NaNO_3_ levitated optically in air using different excitation wavelengths; Figure S2: Visualization of an aqueous micro-droplet of NaNO_3_ optically levitated through the 50X Nikon objective of the confocal Raman microscope and the 100X Nikon inverted immersion objective of the optical tweezer; Video S1: Visualization of the capture process of a particle using the LTRS system developed in this work.
